# State of the art and future directions in assessing the quality of life in rare and complex connective tissue and musculoskeletal diseases

**DOI:** 10.3389/fmed.2022.986218

**Published:** 2022-09-23

**Authors:** Leopoldo Trieste, Sara Cannizzo, Ilaria Palla, Isotta Triulzi, Giuseppe Turchetti

**Affiliations:** Institute of Management, Scuola Superiore Sant’Anna, Pisa, Italy

**Keywords:** daily activity, fatigue, pain, sleep quality, quality of life, rheumatic diseases, PROMS, musculoskeletal diseases

## Abstract

**Background:**

As chronic conditions, rare and complex connective tissue and musculoskeletal diseases (rCTDs) significantly affect the quality of life generating an impact on the physical, psychological, social, and economic dimensions of the patients’ lives, having implications on the family, changing the lifestyle and interpersonal relationships. Traditionally, generic and disease-specific measures for Quality of Life (QoL) provide valuable information to clinicians since QoL affects healthcare services utilization, predicts morbidities and mortalities, workability, etc. Moreover, the assessment of unmet clinical needs, satisfaction, the experience with the treatment and the care, the psychological dimensions, and the effects of the diseases, such as fatigue, could represent valuable dimensions to be considered in the QoL impact assessment. It is also necessary to measure the impact of rCTDs by considering the perspectives of family members/informal caregivers, for instance considering values, beliefs, experiences, life circumstances, psychological aspects, family relationships, economic issues, changes in social activities, etc.

**Objective:**

The aim of this scoping review is to better understand the status of QoL metrics used in clinical and economic research for the assessment of the individual’s perspective on living with rCTDs.

**Research question:**

What are the main challenges in QoL measures (and/or) measurement/assessment in rCTDs?

**Materials and methods:**

Scoping review of the literature referring to QoL measures in rCTDs. Database: PUBMED, ISI-Web of Science; last date: 21/09/2021.

**Results:**

Anxiety and depression, body image satisfaction, daily activity, fatigue, illness perception, pain, personality, QoL, resilience, satisfaction with the relationship, self-management, sexual QoL, sleep quality, social support, stress, uncertainty, and work productivity are the observed dimensions covered by the included studies. However, “more shadows than lights” can summarize the review’s outcome in terms of Patient Reported Outcome Measures (PROMs) domains covered for each of the rCTDs. Also, for those diseases characterized by a relatively high prevalence and incidence, such as Systemic Lupus Erythematosus, Sjögren’s Syndrome, and Systemic Sclerosis, the analysis of patients’ resilience, satisfaction with the quality of the relationship, personality, and stress are still missing dimensions. It has been observed how reducing items, increasing the number of domains, and disease-specific questionnaires characterize the “technological trajectory,” such as the evolution of questionnaires’ characteristics for assessing QoL and QoL-related dimensions and the burden of rCTDs.

**Conclusion:**

The scoping review presents an overview of studies focused on questionnaires used to evaluate the different dimensions of quality of life in terms of general instruments and disease-specific questionnaires. Future research should include the co-design with patients, caregivers, and patient representatives to create questionnaires focused on the unmet needs of people living with rCTDs.

## Introduction

Rare and complex connective tissue and musculoskeletal diseases (rCTDs) are a heterogeneous group of immune-mediated inflammatory diseases. With heterogeneous symptoms, these diseases may affect various organs of the body and, at the latest stage, come to multiple organ systems impairment. rCTDs include diseases such as Systemic Lupus Erythematosus (SLE), Systemic Sclerosis (SSc), Mixed Connective Tissue Diseases (MCTD), Inflammatory Idiopathic Myopathies (IIM), Undifferentiated Connective Tissue Diseases (UCTD), Antiphospholipid Syndrome (APS), IgG4, Sjögren’s Syndrome (SS), Ehlers Danlos (EDS), and Relapsing Polychondritis (RP) (1). The accurate incidence and prevalence of each disease (2) are still undetermined; however, most rCTDs affect no more than 1 person per 2,000 (3), on average (meeting criteria defined by the European Union Regulation on Orphan Medicinal Products 1999), with few exceptions. Despite these diseases being characterized by a variegate spectrum of clinical manifestations (3) with often unpredictable courses that reduce health and wellbeing (4), their clinical and non-clinical burden is scarcely investigated. There are, of course, some exceptions, especially on SLE. It is the case of a comprehensive and recent survey that shows how SLE affected several spheres of patients’ life, in particular, education (50.7%), career (57.9%), and emotional/sexual life (38.2%) (5). Also, Shi et al. (6) showed that disease activities and organ damage are negatively correlated with HRQoL. Moreover, it has been shown that the cognitive impairments were negatively associated with social role and QoL (7) by Mendelsohn et al. Patients with SS are affected by physical and psychological disorders, such as digital ulcers, skeletal muscle weakness, fatigue, and depression, which impair working and social activities (8). Less investigated are family members’ and informal caregivers’ stress. Finally, poor and not detailed evidence on the economic implications and impacts of rCTDs on the sustainability of healthcare systems is available.

With the aim of reducing patients’ symptoms and disability and improving the Health-Related Quality of Life (HRQoL), clinicians are assessing the impact of selected specific symptoms on patients’ functional disability and QoL, and the factors associated with it (9, 10). On this, Patient Reported Outcome Measures (PROMs) are offering no available generic-and disease-specific instruments as in the case of the 36-item Short-Form Health Survey (SF-36) and the Lupus Quality of Life LupusQoL. HRQoL is the most explored dimension among PROMs (10). However, literature on PROMs in rCTDs is still scarce and instruments are characterized by great heterogeneity and applied to small sample sizes (11).

However, to the best of our knowledge, previous systematic reviews investigating the HRQoL of patients with Systemic Lupus Erythematosus (6–9, 12–17), Systemic Sclerosis (18–28), Ehlers-Danlos syndromes (29, 30), Sjögren’s Syndrome (31), and Idiopathic Inflammatory Myopathy (32) focused on the effect of new or existing therapies, and/or the burden of specific diseases on specific categories of patients. Studies included in the reviews adopted PROMs and related questionnaires as tools. It is also the case of those reviews related to pediatrics and adolescents (29), women (33), specific geographic areas and country (34), and also those papers that review the existing economic burden and health resource consumption (35).

Compared to the previous studies, this scoping review gave an overview of the tools (PROMs and QoL) adopted for investigating whether and how the scientific literature has measured the different dimensions of PROMs in rCTDs. To enhance our understanding of the global burden of rCTDs, in fact, there is an urgent need of reviewing all the instruments adopted, e.g., PROMs, and for each of the involved rCTDs, identifying which areas are still unexplored. This scoping review offers to clinicians and researchers a taxonomy of where we are in terms of existing tools for assessing dimensions of QoL, then it indicates the not investigated directions we should follow for a better understanding of the complexity of the non-clinical impact of different rCTDs.

## Materials and methods

### Study design and search strategy

To achieve the scope of the review, we adopted the PICO (Patient/Population Intervention Comparison Outcomes) framework to guide us in part for selecting the minimum quality and available information for including papers and to determine the boundaries of the scoping review; in part, for identifying some of the variables adopted for classifying the included paper (36).

Although in scopes, perspectives, outcomes, and suggestions, this paper is a scoping review, we adopted, as a searching and review method, the more rigorous PRISMA approach (37).

One author performed the literature research and three reviewers, working independently, screened all the studies, and extracted data using a template developed specifically for this study.

#### Research queries

**FIGURE 1:**
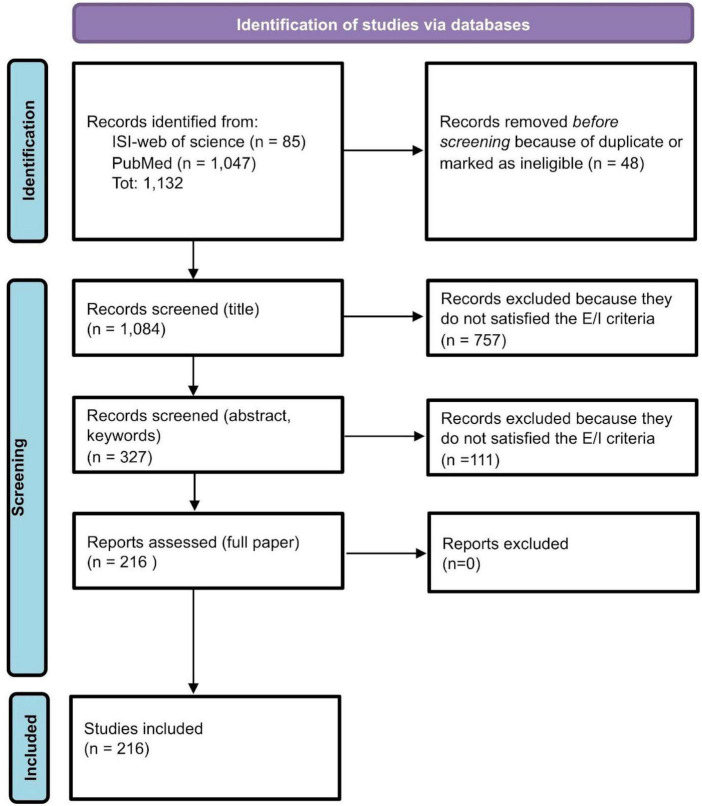
Review process: PRISMA flow-chart (37).

For each database (PubMed and ISI-Web of Science), the research query consists of the intersection of two main fields: words related to QoL and other PROMs (125,143 records from PubMed and 19,497 from ISI-Web of Science); words related to rCTDs diseases (50,316 records from PubMed and 54,759 from ISI-Web of Science) for a total of 1,047 records from PubMed and 85 from ISI-Web of Science. The review process started with these records (see Figure 1). Research queries for the two databases are reported in the Supplementary material.

#### Including/excluding criteria

We included peer-review papers published between 2011 and 2021 in English. Within this time interval, non-English written papers, reports, reviews, theoretical and/or position papers without empirical analysis, and/or presentation of new questionnaires without empirical validation (see classification criteria), as well as papers with physical or clinical endpoints only and outputs of clinical studies have been excluded.

#### Statistics and test

Kruskal-Wallis test *p*-value and *Chi-square* test *p*-value tested median differences among groups of continuous variables and frequency tables of discrete variables, respectively.

#### Classification criteria for the included papers

Papers have been classified according to the type of studies, authors, year, title, journal, country, sample sizes, disease, questionnaire adopted, number of citations (from Google Scholar updated on May 20, 2022), and average number of citations per year. Types of studies have been classified as follows:

A. Studies that assess and compare the impact of new treatments, drugs, interventions, etc., in which QoL and psychological changes are adopted as criteria for assessing the efficacy of the proposed solutions.
B. Methodological studies that are devoted to:
  **B1**: Validate existing metrics, questionnaires, and tools in specific populations and/or countries and/or compare existing questionnaires (e.g., mapping disease-specific vs. generic QoL questionnaires);
  **B2**: Validate new metrics, questionnaires, and tools for assessing QoL and the psychological burden of rare diseases.
C. Studies that identify correlates of QoL and psychological burden with respect to the disease characteristics as ancillary analysis within specific studies. These studies can be analyses performed starting from A, through RCT, observational and interventional, and cohort studies.
D. Epidemiological studies including the socio-economic and psychological dimensions of the diseases, analyses of correlates of QoL, and psychological burden with respect to the disease characteristics in a specific population are included here.

#### Questionnaires classification

Questionnaires included in the paper have been classified according to the scope, number of dimensions, items, and disease specificity.

### Outcomes

After the description of the number of studies selected and included in the scoping review through a PRISMA scheme (see subsection titled “Review process” and Figure 1), outcomes have been divided into three main topics.

#### Bibliometric analysis

**FIGURE 2:**
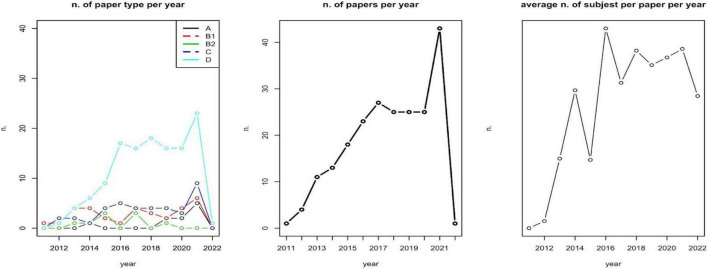
Papers’ type, no. of papers, and average number of involved subjects per year.

We first assessed the relationship between the number of subjects included and the type of papers; the number of involved subjects and the selected diseases; type of disease and type of paper involved. A first analysis describes the number of papers per type per year, the number of papers per year, and the average number of subjects involved (sample size) per year (Figure 2). These analyses aim at identifying an over year positive trend in sample size, and a focalization on methods and approaches for assessing patients’ PROMs.

**FIGURE 3:**
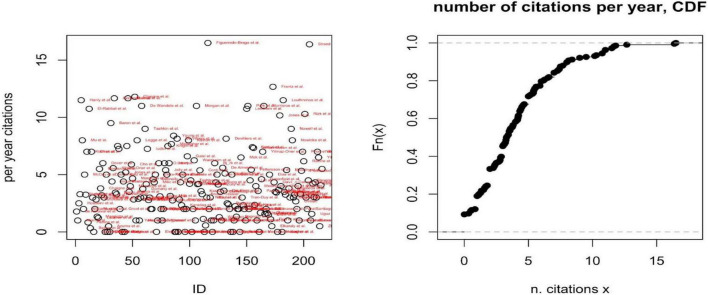
Per year number of citations (left) and cumulative distribution function (CDF) of the number of citations per year (right).

A second descriptive analysis reports the number and distribution of citations per year (Figure 3), as a proxy of the field and specific paper’s impacts on the scientific community. A third analysis reports the per year most cited paper per included disease and PROMs’ domain (Table 1).

**TABLE 1:** Most cited and relevant papers per patient reported outcome domain and disease.

	FM	APS	AS	CLE	DM	EDs	IID	IIM	IMIDs	IRDs	LN	RA	RDs	SLE	SS	SSc	OP	UCTD	MPYC
Anxiety and dep.	Nowell et al. (46)	0	0	0	0	De Wandele et al. (47)	0	0	0	0	0	Figueiredo-Braga et al. (38)	0	Figueiredo-Braga et al. (38)	Koçer, (48)	Sariyildiz et al. (49)	0	0	Figueiredo-Braga et al. (38)
Body image sat.	0	0	0	0	0	0	0	0	0	0	0	0	0	0	0	Mills et al. (39)	0	0	Mills et al. (39)
Daily activity	0	0	0	0	0	0	0	0	0	0	0	0	0	Rizk et al. (50)	Rizk et al. (50)	Tashkin et al. (51)	0	0	Rizk et al. (50)
Fatigue	0	0	0	0	Dover et al. (52)	De Wandele et al. (47)	0	0	0	0	0	Figueiredo-Braga et al. (38)	0	Mahieu et al. (53); Azzizodin et al. (54)	Priori et al. (55)	0	0	0	Figueiredo-Braga et al. (38)
Illness perception	0	0	0	0	0	0	0	0	0	0	0	0	0	Nowicka et al. (56)	0	Frantz et al. (10)	0	0	Frantz et al. (10)
Mixed	0	Nowell et al. (46)	Nowell et al. (46)	0	0	0	0	Armadans-Tremolosa et al. (57)	0	0	0	Nowell et al. (46)	0	Mahieu et al. (53)	McCoy et al. (58)	Morrisroe et al. (59)	Nowell et al. (46)	0	Mahieu et al. (53)
Pain	0	0	0	0	0	Muriello et al. (60)	0	0	0	0	0	0	0	Jones et al. (61)	0	Cengiz et al. (62)	0	0	Jones et al. (61)
Personality	0	0	0	0	0	0	0	0	0	0	0	0	0	0	Milic et al. (40)	0	0	0	Milic et al. (40)
QoL	Uguz et al. (63)	Zuily et al. (64)	Chen et al. (65)	Samotij et al. (66)	Dover et al. (52)	De Wandele et al. (47)	Xu et al. (67)	0	Spierings et al. (68)	Haglo et al. (69)	Jolly et al. (70)	Figueiredo-Braga et al. (38)	Moorthy et al. (71)	Strand et al. (16)	Park et al. (45)	Frantz et al. (10)	0	Iudici et al. (72)	Figueiredo-Braga et al. (38)
Resilience	0	0	0	0	0	0	0	0	0	0	0	0	0	Li et al. (41)	0	0	0	0	Li et al. (41)
Rel. satisfaction	0	0	0	0	0	0	0	0	0	0	0	Figueiredo-Braga et al. (38)	0	Figueiredo-Braga et al. (38)	0	0	0	0	Figueiredo-Braga et al. (38)
Self-management	0	0	0	0	0	0	0	0	0	0	0	0	0	0	0	Mattsson et al. (42)	0	0	Mattsson et al. (42)
Sexual QoL	0	0	0	0	0	0	0	Heømánková (73)	0	0	0	0	0	0	Priori et al. (55)	0	0	0	Priori et al. (55)
Sleep	0	0	0	Samotij et al. (66)	0	Domany et al. (74)	0	0	0	0	0	Figueiredo-Braga et al. (38)	0	Mirbagher et al. (75)	Chung et al. (76)	Sariyildiz et al. (49)	0	0	Figueiredo-Braga et al. (38)
Social support	0	0	0	0	0	0	0	0	0	0	0	0	0	Zeng et al. (43)	0	0	0	0	Zeng et al. (43)
Stress	0	0	0	0	0	0	0	0	0	0	0	0	0	Morgan et al. (44)	0	0	0	0	Morgan et al. (44)
Uncertainty	0	0	0	0	0	0	0	0	0	0	0	0	0	Li et al. (41)	0	0	0	0	Li et al. (41)
Work productivity	0	0	0	0	0	0	Xu et al. (67)	0	0	0	0	0	0	Kernder et al. (77)	0	Morrisroe et al. (59)	0	0	Morrisroe et al. (59)

MPYC: paper with the max per year citations. Diseases: FM, Fibromyalgia; APS, Antiphospholipid, antibodies Syndrome; AS, Ankylosing spondylitis; CLE, Cutaneous lupus erythematosus; DM, Dermatomyositis; EDs, Ehlers–Danlos syndrome; IID, Immunoinflammatory diseases; IIM, Idiopathic inflammatory myopathies; IMIDs, Immune-mediated inflammatory diseases; IRDs, Inflammatory rheumatic diseases; JDM, Juvenile dermatomyositis; LN, Lupus nephritis; RA, Rheumatoid arthritis; RDs, Rare diseases; SLE, Systemic lupus erythematosus; SS, Sjögren’s syndrome; SSc, Systemic sclerosis; OP, Osteoporosis related to rheumatic disease; UCTD, Undifferentiated Connective Tissue disease. Acronyms in bold collect different diseases.

#### Analysis of questionnaires

**FIGURE 4:**
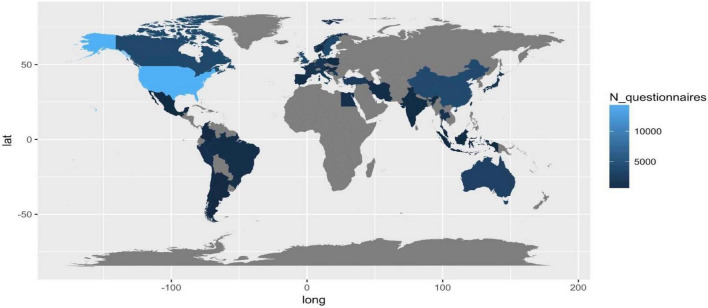
Total number of questionnaires administered per country.

It consists of three main analyses. First, we reported the number of total questionnaires administered per country, then, we reported the list of questionnaires adopted with indications of their domains and total items (Table 2). Finally, focusing on the most frequently covered dimensions of PROMs (i.e., anxiety and depression, fatigue, pain, and QoL), we analyzed the trend over the year of the related number of items, the number of domains, and the disease-specificity of questionnaires, to highlight a “technological” trajectory of the tools and metrics adopted for assessing patient’s PROMs (Figure 4).

**TABLE 2:** Classification of questionnaires adopted per scope, no. of domains, no. of issues, and disease specificity.

Acronym	Name	N of domains*	N of issues	DS (Yes = 1, No = 0)	Acronym	Name	N of domains*	N of issues	DS (Yes = 1, No = 0)

Anxiety and depression and psychological dimensions					QoL and related issues				
BAI	Beck anxiety inventory	21	21	0	PSS-QoL	Health-related quality of life in PSS patient	25	25	1
GAD7	General anxiety disorder	7	7	0	QOLS	Quality of life scale	5	15	0
HADS	Hospital anxiety and depression scale	14	14	0	QoML	Quality of my life	-	-	0
HAM	HAMILTON ANXIETY RATING SCALE	14	14	0	SF-12	Short-form twelve dimensions	8	12	0
Herth	Herth hope index	12	12	0	SF-36	Medical outcomes study short form-36	8	36	0
MDAS	Modified dental anxiety scale	5	5	0	SF-6D	Short-form six-dimension	6	6	0
MHISS	Mouth handicap in systemic sclerosis scale	3	12	1	SHAQ	Scleroderma health assessment questionnaire	4	23	1
PSC	Pediatric symptom checklist	35	35	0	SLEQoL	Systemic lupus erythematosus quality of life questionnaire	6	40	1
SCARED	Screen for child anxiety related disorders	5	41	0	SSC	SLE symptom checklist	38	38	1
ZR-SAS	Zung self-rating anxiety scale	20	20	0	SSCQoL	Systemic sclerosis quality of life scale	4	29	1
BDI	Beck depression inventory	21	21	0	V-QoL	Voice-related quality of life	4	10	0
CES-D	Center for epidemiologic studies depression scale	20	20	0	WHOQOL	World health organization QoL group questionnaire	4	26	0
HDRS	Hamilton depression rating scale	17	17	0	SMILEY	Simple measure of impact of lupus in youngster	4	24	1
ZSR-ADS	Zung self-rating anxiety depression scale	-	-	0	CHAQ	Childhood health assessment questionnaire	8	8	0
TSK	Tampa scale of kinesiophobia	17	17	0	QL-Index	Quality of life index	-	-	0
SCID	Structured clinical interview	10	10	0	SarQoL	Sarcopenia quality of life	7	22	1
**Fatigue**					PGI	Patient-generated index	-	-	0
FACIT-F	Functional assessment of chronic illness therapy-fatigue	40	40	0	**Sleep quality and related issues**				
FSS	Fatigue severity scale	9	9	0	ISI	Insomnia severity index	7	7	0
MFI	Multidimensional assessment of fatigue	5	20	0	PSQI	Pittsburgh sleep quality index	7	19	0
CIS	Checklist individual strength measures	4	20	0	SBMI	Symptom burden index	8	8	0
**Pain and related issues**					**Sexual QoL**				
ASESP	Arthritis self-efficacy scale pain and other symptoms subscale	3	20	0	SQoL-F	Sexual quality of life questionnaire–female	5	18	0
BPI	Brief pain inventory	2	11	0	SQoL-M	Sexual quality of life questionnaire–male	5	11	0
MPQ-SF	McGill pain questionnaire SF	2	15	0	FSFI	The female sexual function index	6	19	0
PD-Q	Pain detect questionnaire	1	12	0	**Stress**				
PFSD	Pain-frequency-severity-durationscale	5	5	0	PSS	Perceived stress scale	1	10	0
PHQ	Patient health questionnaire	9	9	0	STAI	The state-trait anxiety inventory	2	80	0
PIVAS	Pain intensity visual analog scale	-	-	0	**Self-management**				
PCQ	Pain coping questionnaire	39	8	0	PAM	Patient activation measure	-	-	0
FPS-R	Faces pain scale-revised	-	-	0	SEMCD	Self-efficacy for managing chronic diseases	6	6	1
**QoL and related issues**					**Others**				
EQ-5D	EuroQoL-5 dimensions/Visual analog scale	5	5	0	IPQ	Illness perception questionnaire	9	9	0
KBILD	Kings’s brief interstitial lung disease questionnaire	3	15	1	CD-RISK	Connor-Davidson resilience scale	5	25	0
LCQ	Leicester cough questionnaire	19	19	1	HAQ	Health assessment questionnaire	8	20	0
LIT	Lupus impact tracker	10	10	1	MUIS-A	Mishel uncertainty in illness scale for adults	5	30	0
LupusPRO	Lupus patient-reported outcome TOOL (LupusPRO)	3	43	1	NEO-P-R-I	Revisited NEO personality inventory	5	240	0
LupusQol	Disease-specific health-related quality of life measure for adults with SLE	8	34	1	RAS	Relationship assessment scale	7	7	0
NEURO-QoL	Neurological health related quality of life	13	13	0	SEMCD	Self-efficacy for managing chronic diseases	6	6	1
NHP	Nottingham health profile	7	45	0	SSRS	Social support rating scale	3	10	0
OHIP-14	Oral health impact profile	7	14	0	SWAP	Satisfaction with appearance scale	14	14	0
OHIP-49	Oral health impact profile	7	49	0	WPAI	Work productivity and activity impairment	6	6	0
PedsQL	Pediatric quality of Life inventory	4	23	0	PROMIS	Patient-reported outcomes	4	29	0

*Explicitly declared. If number of items = number of domains- > domains are not explicitly declared. DS, disease specificity.

#### Dimensions on patient reported outcome measures covered per disease

It is the core and the main outcome of the present scoping review. Each row of Table 3 refers to a specific PROM dimension over the different diseases. Each column of the table represents, for a specific disease, the dimensions of PROMs covered. Therefore, Table 1 shows the state of the art in our knowledge/ignorance of dimensions that describe how the selected diseases impact patients.

**TABLE 3:** Patient reported outcome measures (PROMs’) dimensions explored for each disease.

	FM	APS	AS	CLE	DM	EDs	IID	IIM	IMIDs	IRDs	LN	RA	RDs	SLE	SS	SSc	OP	UCTD	Tot.
Anxiety and dep.	1					2						2		32	13	3			53
Body image sat.																1			1
Daily activity														1	1	6			8
Fatigue					1	1						1		20	5				28
Illness perception														1		1			2
Mixed		1	1					1				1		8	2	3	1		18
Pain						3								10		2			15
Personality															1				1
QoL	2	3	3	2	6	6	1		1	1	3	12	1	130	26	36		1	234
Resilience														1					1
Rel. satisfaction												1		1					2
Self-management																1			1
Sexual QoL								2							1				3
Sleep						1						2		10	3	1			17
Social support														1					1
Stress														2					2
Uncertainty														1					1
Work productivity							1							2		1			4
**Tot**.	3	4	4	2	3	13	2	3	1	1	3	19	1	220	52	55	1	1	392

Diseases: FM, Fibromyalgia; APS, Antiphospholipid, antibodies Syndrome; AS, Ankylosing spondylitis; CLE, Cutaneous lupus erythematosus; DM, Dermatomyositis; EDs, Ehlers–Danlos syndrome; IID, Immunoinflammatory diseases; IIM, Idiopathic inflammatory myopathies; IMIDs, Immune-mediated inflammatory diseases; IRDs, Inflammatory rheumatic diseases; JDM, Juvenile dermatomyositis; LN, Lupus nephritis; RA, Rheumatoid arthritis; RDs, Rare diseases; SLE, Systemic lupus erythematosus; SS, Sjögren’s syndrome; SSc, Systemic sclerosis; OP, Osteoporosis related to rheumatic disease; UCTD, Undifferentiated Connective Tissue disease. Acronyms in bold collect different diseases. Anxiety and dep., anxiety and depression; Body image sat, body image satisfaction; Rel. satisfaction, satisfaction with the relationship. Totals do not correspond to the number of papers since there are cases of more than one disease, and more than one questionnaire per area assessed in the same paper.

## Results

### Review process

After removing duplicates or ineligible papers because of the lack of abstracts and essential information, 1,084 papers’ abstracts from ISI-Web of Science and PubMed have been screened. A total of 327 papers survived the process. After excluding 111 articles because out of the objective of our review, we fully assessed 216 papers. All 216 papers have been included and classified in the present review (see Figure 1).

### Bibliometric analysis

The majority of included papers are of type D (A: 4.63%; B1: 14.81%; B2: 4.17%; C: 17.59%; D: 58.80%). There is a non-significative positive relationship between the number of subjects included and A–D classifications (Kruskal-Wallis test *p*-value = 0.1699). The number of involved subjects also depends on the selected diseases (Kruskal-Wallis test *p*-value = 0.027). No effect of disease on the type of paper has been observed (*Chi-square* test *p*-value = 0.498).

As reported in Figure 2, during the years 2019–2020, a zero-growth rate can be observed in the number of scientific papers published as well as the number of subjects involved per paper (on average). A deeper and specific analysis could be carried out for identifying whether this is caused by the COVID-19 pandemic.

#### Diseases involved and questionnaires

A total of 7.8% of the included papers analyze/compare two diseases, 4.2% analyze/compare three diseases, 3.2% analyze/compare four diseases, and 0.46% analyze/compare five, six, and seven diseases. A total of 48.61% of the included papers’ outcomes are based on two validated questionnaires, 22.22% on three, 8.79% on four, and 5.09% on five validated questionnaires for assessing QoL and/or QoL-related dimensions and PROMs.

#### Impact of studies on the scientific community

The number of citations per year has been adopted as a proxy of the paper’s impact on the scientific community. The cumulative distribution function of the number of per year citations shows how the impact of each paper is relatively low, on average (i.e., the probability that a paper is cited at least five times per year is only 0.3); only two papers are cited more than 15 times per year, on average (Figueiredo-Braga et al. and Strand et al.), both focusing on SLE. For each disease and dimensions of PROMs, Supplementary material report the most cited or the only paper included.

#### Most cited papers per patient reported outcome measures’ domain and disease

Table 1 reports the most cited or the only available article per PROM’s domain and disease. The reported papers can be adopted as benchmarks in the covered field. Also, on the list of the most cited papers, there are some contributions that cover more than one PROM dimension and more than one disease, i.e., they recur on the table cells both horizontally (by PROMs dimension) and vertically (by disease). There are papers with high impact (measured in per year number of citations) with respect to PROMs and diseases covered. It is the case of Figeiredo-Braga et al. (38), in SLE and Rheumatoid arthritis (RA), that assesses anxiety and depression, QoL, satisfaction with the relation, and sleep quality. The last column of Table 1 collects the per-year most cited papers per PROM dimension, on the most cited papers per PROM and disease. With respect to dimensions that are not often covered, Mills et al. (39) investigate body image satisfaction for SSc patients; Milic et al. (40) analyze the personality of SS patients; Li et al. (41) investigate resilience in SLE; Mattsson et al. (42) analyzed self-management and sexual QoL (SS patients); Zeng et al. (43), Morgan et al. (44), and Park et al. (45) analyzed social support, stress, uncertainty in SLE patients, respectively.

### Analysis of questionnaires

#### Administered questionnaires/subjects involved per countries

Considering the number of total questionnaires administered per county as a proxy of interest in PROMs related to the included disease, we observe the lack of information in Russia, South-Russia countries, Central America, Africa, and Arabia. The quality and efficiency of healthcare systems and data (e.g., USA, Canada), and the incidence and prevalence of country-specific diseases (e.g., Turkey and Iran) can explain the high frequency of studies in specific regions.

#### Technological trajectories of questionnaires

Treating questionnaires as “technologies” for assessing PROMs, three drivers guide their design: number of domains (D), number of issues (I), and disease specificity (DS).

#### Disease specificity

**FIGURE 5:**
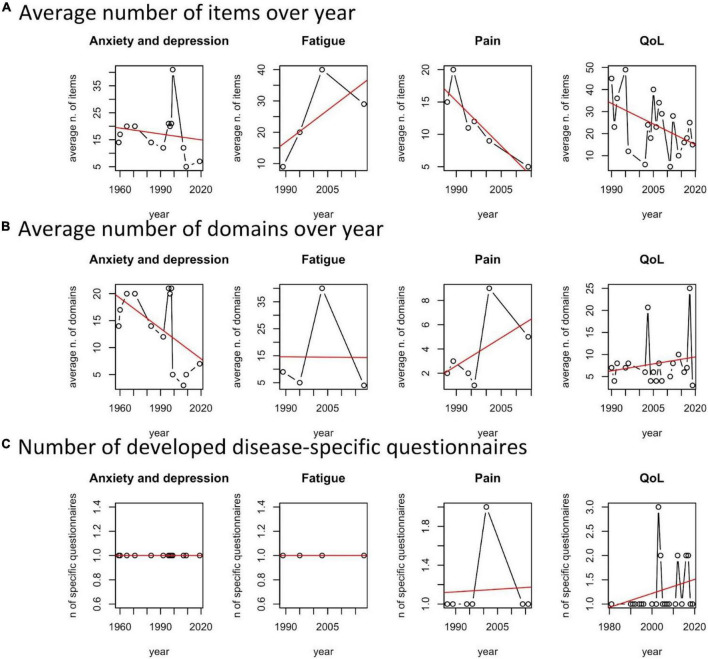
Average number of issues, domains, and number of developed disease-specific questionnaires per year and patient reported outcome measures (PROMs) area.

We observed a moderate tendency of increasing disease specificity in QoL and Pain questionnaires (Figure 5C).

#### Number of domains and issues

With respect to the average number of issues of the developed questionnaires per year and scope, we noted a general tendency of improving the survey’s efficiency by cutting the number of questions. This is observed for validated questionnaires on anxiety and depression, pain and related issues, and QoL (Figure 5A). There is a tendency of increasing the number of domains for Pain and QoL (Figure 5B). The exception is the domain “fatigue,” probably because of increasing awareness of scholars on the different dimensions involved in describing it.

To summarize, reduction of items, increasing number of domains, and disease-specific questionnaires characterize the “technological trajectory” of questionnaires for assessing QoL and QoL-related dimensions and the burden of rCTDs.

### Diseases and dimensions of patient reported outcome measures covered

Table 3 reports the number of papers that adopted validated questionnaires for assessing the dimensions of the disease. Black cells indicate that, for a particular disease, a specific dimension is not covered.

As one can observe, only questionnaires on QoL are frequently adopted for many of the involved diseases. pSS, RA, SLE, SS, and SSc are diseases for which we have more information on anxiety and depression, illness perception, pain, stress, and social support (especially for SLE). Also, considering the most studied diseases because of a relatively higher number of available patients to be enrolled, body image satisfaction and self-management are not assessed for SS, RA, SLE, and SS. It is also the case for illness perception in SS and RA (in general). Social support is covered only for SLE. The less covered dimensions, in general, and among the different diseases are body image satisfaction, resilience, and the assessment of personality. These are no less important dimensions, influencing medical-patient interactions and communication, adherence, behavioral changes, and the effect of social marketing initiatives, as well as the efficacy of therapies and treatments.

## Discussion

The state of the art on QoL and other PROMs related to rCTDs suggests five areas of challenges and area of future research.

First, there are diseases that need to be investigated beyond the generic questionnaires on QoL (e.g., APS, Antiphospholipid, antibodies syndrome; AS, Ankylosing spondylitis; JDM, Juvenile dermatomyositis; LN, Lupus nephritis).

Second, there are dimensions that are not covered for some relatively well-investigated diseases, e.g., stress, satisfaction with the relation, and resilience. We wonder if, over the years, Table 3 of our review will reduce the number of black cells, also for those diseases that are not in the current spotlight as it happens for SLE and SSc.

Third, Table 3 collects dimensions that are covered at least one time over the included papers and disease. Therefore, there are dimensions still not covered by the current literature, such as prescription and therapy compliance, family organization’ changes induced by the disease, etc., and other dimensions that could emerge only through a bottom-up approach and by adopting complementary qualitative methods, such as Narrative Medicine, focus groups, etc.

A fourth emerged theme (see Figure 5) is related to the available and adopted questionnaires for each dimension of PROMs. Although the average number of questions per questionnaire is falling in a specific domain like QoL, SF-36 is the standard, the heterogeneity of the adopted questionnaires and the lack of specific mapping among the related questionnaires make it very difficult to offer a valid suggestion for selecting the most appropriate questionnaire, neither to summarize too heterogeneous scores into a general synthesis. This mainly depends on the lack of a general consensus on the meaning of quality of life among scholars. Mapping scores of different questionnaires could fill the current complexity and uncertainty, supporting researchers in selecting the most appropriate questionnaires covering different dimensions of PROMs.

Finally, although it is a fundamental topic in improving the sustainability, quality, and equity of healthcare systems, no evidence emerged on the relationship between QoL and the other dimensions of PROMs, on one side, and Patient Reported Experience Measures (PREMs) on the quality of existing and new healthcare service delivery, both from the perspective of patients and healthcare providers, on the other side. Mapping validated questionnaires related to PROMs and PREMs could improve our understanding of patients’ needs and reaction to the offered healthcare services.

Also, specific QoL assessment techniques in rCTDs may fill the gap with respect to well-known diseases. For instance, patients can report their quality of life and experiences in dedicated digital diaries, and/or through specific texts. The techniques adopted in narrative medicine are promising and new dimensions related to the quality of life may rise in the future to come.

Moreover, Big Data could be adopted for detecting and analyzing rCTD patients’ behaviors, quality of life, and decision-making without dedicated questionnaires. However, the role of Big Data is limited by sample sizes and ethical and legal/privacy constraints.

Finally, note that all dimensions reported in the scoping review and those indicated for future investigations refer to subjective and conscious answers coming from the patient. New advanced techniques can also detect positive or negative experiences with care and treatment by means of objective measures of unconscious feelings. Neuroscience and consumer neuroscience techniques and new measures, such as frontal alpha-wave asymmetry (78), detected through electroencephalography (EEG) (79) are promising solutions. However, if they solve biases of the current questionnaires through which conscious answers on *ex post* rationalized experiences are collected, these interesting and new techniques are not able to overcome and usually increase the problem of insufficient sample sizes.

## Strength and limitations

The strength of the current scoping review is in the number of included and classified papers (216), on the capability to check, for disease and PROMs domain, and the availability or lack of studies and instruments. Since the scoping review covers the field of rCTDs, it overcomes the existing methodological reviews that are usually disease specific (9, 78). Also, to our knowledge, this is the first work that quantitatively analyzes “technological” trajectories of questionnaires on the basis of the number of items, number of domains, and disease specificity.

However, this scoping review has some limitations and weaknesses. First, although it mentions the most cited papers, it does not report their outcome. As clarified in the introduction, the review can answer whether and how some dimensions have been covered but not what and the relation with the clinical outcomes. Since it was not the aim of our scoping review, scholars and/or professionals that are interested in dimensions not covered in the present work can refer to disease-specific reviews. Second, our analysis is limited to the specific questionnaires adopted and used and it does not refer to all the available questionnaires and PROMs. Third, specific analyses on questionnaire subdomains have not been conducted. As a consequence, we did not take into account other questionnaires’ subdomains that refer to the selected PROMs. Fourth, we focus our attention on disease-specific vs. generic questionnaires. Therefore, although Table 2 reports population-specific questionnaires (e.g., questionnaires for pediatric patients), we did not conduct analysis on this topic. Fifth, with respect to the number of questionnaires administered per country, we did not detail data for a specific disease but we referred to the general field of rCTDs. Finally, the time-interval selection is not justified by some discoveries or facts that justify the adoption of a 10-year period for including/excluding papers. Fixing a “related to the field” factor for selecting time-interval would have shifted the objective of the review, from assessing how QoL and the related dimensions are covered, to assess the impact of the specific factor (e.g., a new drug and a new specific questionnaire) on the assessment of QoL and the other dimensions for rCTDs patients.

## Conclusion

This scoping review presents an overview of studies focused on questionnaires used to evaluate the different dimensions of quality of life in terms of general instruments and disease-specific questionnaires. Future research should include the co-design with patients, caregivers, and patient representatives to create questionnaires focused on the unmet needs of people living with rare and complex connective tissue and musculoskeletal diseases.

## Author contributions

SC was responsible for the manuscript’s abstract and keywords. IT and LT were responsible for the manuscript introduction. SC and LT edited methods. LT, IP, and IT reviewed and classified the included manuscripts. LT quantitatively analyzed the classified manuscript and edited results, tables, and figures in results. All authors contributed equally to the discussion and conclusion, and also revised the final version of the manuscript and the Supplementary material.
